# The Nuclear Transcription Factor CREB: Involvement in Addiction, Deletion Models and Looking Forward

**DOI:** 10.2174/157015907781695937

**Published:** 2007-09

**Authors:** Cameron S McPherson, Andrew J Lawrence

**Affiliations:** 1 Brain Injury and Repair Group, Howard Florey Institute, University of Melbourne, Parkville, Victoria 3010, Australia; 2 Centre for Neuroscience, University of Melbourne, Parkville, Victoria 3010, Australia

**Keywords:** CREB, conditional knockout, addiction, cAMP response element binding protein, behavior.

## Abstract

Addiction involves complex physiological processes, and is characterised not only by broad phenotypic and behavioural traits, but also by ongoing molecular and cellular adaptations. In recent years, increasingly effective and novel techniques have been developed to unravel the molecular implications of addiction. Increasing evidence has supported a contribution of the nuclear transcription factor CREB in the development of addiction, both in contribution to phenotype and expression in brain regions critical to various aspects of drug-seeking behaviour and drug reward. Abstracting from this, models have exploited these data by removing the CREB gene from the developing or developed mouse, to crucially determine its impact upon addiction-related processes. More recent models, however, hold greater promise in unveiling the contribution of CREB to disorders such as addiction.

## INTRODUCTION

The compulsive or uncontrolled use of a drug is often used to define addiction [57], which develops after repeated drug exposure, despite severe adverse consequences [58, 79]. It is the progression from recreational or controlled use of a drug to this unbalanced, compulsive state that is a distinguishing hallmark of addiction. The underlying pathology of addiction has been partly described by different theories, and prominently includes the opponent process theory [57, 109], an incentive salience model explaining excessive drug wanting [9, 53, 97], and the development of learned adaptations, describing drug-memory associations [22]. Unifying these theories is a compelling, underlying proposal that the development of addiction relates to long term drug-induced neural adpatations, some of which may be manifest following even an acute drug exposure. Such adaptations are observed through behavioural testing and include dependence (where compensation for drug effects beget withdrawal-symptoms subsequent to cessation of drug intake) and tolerance (a condition where drug effects diminish subsequent to ongoing drug exposure [55]). In contrast, sensitization represents a phenomenon involving enhanced drug response, typically subsequent to a cycle of drug exposure and abstinence [9, 22, 53, 97]. Moreover, the contextual association between drug and environment dramatically impacts upon the development and expression of sensitization [9, 97]. It should be noted, however, that physical dependence upon a substance is not a necessary precondition for addiction. In sum, addiction has been likened to an abberent form of learning, involving stable changes responsible for long-term behavioural plasticity, manifesting as changes in behavioural response to acute or repeated drug exposure [33, 52, 104].

A recurring issue associated with rehabilitated drug addicts is their ongoing potential to relapse, which is not fully ameliorated either by long periods of abstinence nor psychological treatments. This suggests that molecular changes in the brain have been instantiated by a nominal period of drug exposure, not reversible by drug abstinence alone. Berke and Hyman [8] describe this process as a drug-induced usurpation of molecular mechanisms commonly involved with associative learning, which drive compulsive drug abuse and propensity to relapse. Whether molecular or synaptic alterations in neuronal communication or function are a homeostatic adjustment to drug insult or longer term plasticities contributing toward learned behaviours is not clear [8]. Nevertheless, the end result is a complex neural change for which we have so far failed to fully characterise the substrates involved and the specific role that they play. 

Shaywitz and Greenberg [106] make the point that, *“Understanding of the mechanisms by which extracellular stimuli induce changes in gene expression is critical for understanding how cells can adapt to environmental cues.”* Thus, in order to comprehend the technical and complex nature of the behavioural response associated with drug abuse, the apposition of molecular evidence is required to help explain these changes at the cellular level and ultimately, neural systems level. An immediate imperative driving current addiction research is the identification of a molecular target which can mediate long term neuronal lability. ∆FosB [73, 82] and PSD-95 [124] alike have been recently identified as leading substrates in this regard, with modified expression observed up to four and eight weeks following drug stimulus respectively. Moreover, chronic cocaine has been associated with increases in dendritic spine density in the NAcc, but these changes do not appear long-lived enough to correspond with prolonged behavioural changes [81]. Accordingly therefore, other potential molecular targets implicated in the development of addiction are worthy of examination.

It is widely believed that changes in gene expression underlie neural adaptations following exposure to drugs of abuse [28]. A key mechanism for regulating gene expression is through nuclear induction of the transcription factor CREB, implicated also in various affective states, learning and memory. As indicated in Mayr and Montminy [71], *“At a mechanistic level, CREB is perhaps one of the best understood phosphorylation-dependent transcription factors. By comparison, relatively little is known about the physiological role of this protein in different systems.”* This challenge has fallen to, and been taken up by, a surfeit of behavioural, molecular and neuropharmacological investigations, and slowly, a complex picture is emerging. A key challenge for investigators, however, is the advance from a discrete molecular and neuronal synthesis to that of a systems level, as thoroughly emphasised by Nestler [79-81]. Indeed, as Greengard [40] notes, with one hundred billion neurons in the brain, and each sharing approximately one thousand reciprocal connections, deducing the collective afferent and efferent neuronal colloquy is a formidable task. Appreciating the inter- and intra-communications of discrete neural systems comes from understanding their function at a molecular level. 

Here, we examine recent evidence supporting the contribution of CREB as a key molecular mechanism in the development of addiction. Further to this, a variety of models are discussed, which have resolved to restrict or delete the expression of this transcription factor, attempt to explain the role CREB has played. Finally, we comment upon more recent and effective techniques for the restriction of CREB deletion in the adult brain. 

## INVOLVEMENT OF CREB IN ADDICTION

The current state of addiction neurobiology is charactersing a molecular substrate which can partially explain the ongoing behavioural changes wrought by addiction, however, this was not always the case. Earlier work sought to determine which regions of the brain were critically involved in addiction, and subsequently, what pathways were consistently activated. An extensive body of data has now thoroughly demonstrated that the activation of the mesocorticolimbic dopamine system is a key mechanism inovolved in drug reward and reinforcement, a system which involves dopaminergic projections from the mesencephalon that synapse onto the nucleus accumbens, striatum, aymgdala and prefrontal cortex. Studies employing both lesioning experiments [27, 30, 37, 45, 48] and pharmacological manipulations [31, 103, 113] have collectively demonstrated the importance of such structures including the prefrontal cortex (PFC), nucleus accumbens (NAcc), dorsal striatum, hippocampus and amygdaloid nuclei in relation to drug-seeking behaviour and drug-induced plasticity. The activity of this network, and the interface of this network with other structures (e.g. hypothalamus and brain stem nuclei) is critical to long lasting molecular changes driving relapse. 

Dopamine receptors are G-protein coupled receptors (GPCRs) which involve the regulation of adenylate cyclase (AC), generating the second messenger cyclic AMP (cAMP) from ATP. Indeed, it was as early as 1987 that cAMP was found to enhance CREB activation in PC-12 (pheochromocytoma cell line) nuclear extracts [77]. This discovery heralded the identification of a molecular target regulating gene expression which could potentially explain the central development of addiction. Subsequent study investigated other intracellular signal transduction pathways culminating in the activation of CREB, as well as various paradigms of drug abuse which activated this key molecular substrate. As corticostriatal and corticotegmental glutamatergic efferents are also implicated in drug reward, calcium-activated CREB was examined, and the field of study burgeoned. Some of the intracellular signalling transduction pathways implicated in the activation of CREB are demonstrated in Fig. (**1**), and include G-protein, ion channel and growth factor drug receptor targets. These pathways often involve the association of a drug ligand with its respective receptor target, and a kinase phosphorylation cascade culminating in the translocation of a second messenger into the nucleus, phosphorylating CREB at Ser^133^ (pCREB). CREB is a member of a basic leucine zipper (bZIP) CREB/ATF-1 sub-family which includes members CREM (cAMP Responsive Element Modulatory protein) and ATF-1 (Activating Transcription Factor 1), both which bear high bZIP region sequence homology [106], and can bind as homo- or hetero-dimers with these members to the canonical cAMP Responsive Element (CRE) consensus sequence via the leucine zipper in the promoter regions of target genes [11, 22, 61]. The CREB/CREB homodimer exhibits a half-life of 10-20 minutes [106]. Subsequent to phosphorylation at Ser^133^, CRE-bound CREB can exert its influence upon target gene transcription and interact with promoter-bound cofactors. The literature has so far less effectively studied the contribution of family members CREM and ATF, or their various isoforms.

## EVIDENCE FOR CREB IN ADDICTION

An ever increasing body of evidence implicates the molecular actions of CREB in experimental paradigms related to addiction, a small number of which are demonstrated in Table **1**. More recently, studies have examined the expression of pCREB following a variety of drugs of abuse employing *in vivo* models, off a background of over fifteen years of *in vitro* work with cell cultures. Largely, these studies have provided a conflicting array of results regarding positive and negative regulation of pCREB expression, with divergences particulary noted across brain regions examined and temporal follow-up, as well as the period of administration including acute, chronic and subsequent to precipitated withdrawal. Many of these details are demonstrated in Table **1**, in particular the delay to neurochemical analysis as well as treatment paradigm.

Almost unfailingly, natural or precipitated withdrawal from drugs of abuse leads to a dramatic alteration in the expression of nuclear pCREB. Whilst the positive or negative nature of this impact appears dependent upon the brain region studied, substantial pCREB changes have been observed following withdrawal from ethanol (EtOH) [84, 86, 88], morphine [23, 41], psychostimulants (including: amphetamine [46], cocaine [60] and methamphetamine [75], MDMA [70]) and nicotine [15, 85, 94]. This may suggest a regional sensitization in the pCREB response, elsewhere deputised through CRE activation reporters [3, 7, 14, 105], providing a molecular stimulus for relapse behaviour. Together these data suggest that repeated exposure to multiple drugs of abuse may produce sustained activation of intracellular transcription factors, resulting in the persistent and altered expression of functionally important gene products that may underlie the onset and maintenance of addiction [36]. Clearly, a role for pCREB in this process is beyond doubt, and it remains to us as investigators to fully unlock and characterise the methods and higher-level mechanics behind its action. 

## THE DIVIDE BETWEEN CREB AND pCREB

Of those papers listed in Table **1** and elsewhere [125] which examined the impact of drug upon nuclear CREB expression, few of them demonstrated some corresponding shift in its expression levels [74, 85, 94]. In contrast, substantial variation in the expression pattern of its activated form, pCREB, was observed. Such observations were made consistently across discrete brain regions and other physiologically significant organs, revealing little correlation between CREB and pCREB levels. It may be that CREB levels are more invariant to change given the necessary involvement gene transcription, translation and subsequent post-translational modification events, whereas, the changes in pCREB levels are merely indicative of protein kinase action. 

A good reason for differential CREB or pCREB expression is that different central nuclei and cell populations offer various gene targets for these transcription factors, each likely exhibiting their own sensitivites to changes in the CREB transcription factor to induce gene transcription. Mayr and Montminy [71] tabulate some 105 genes with functional CRE motifs identified in the literature, half containing a single CGTA motif. One quarter of these CRE-bearing genes function in cellular metabolism, and the vast majority in some way could directly contribute to neural adaptation or synaptic plasticity following drug insult. Whilst a small number of CRE-bearing genes endogenous to the brain have been identifed in the literature, this observation underlies the likelihood that CREB is likely to impact differently in various neural regions. In a recent review, Carlezon and colleagues [16] highlight that CREB gene promoter targets number in their thousands and that not all CRE motif containing genes are functional CREB targets. In their words, *“the consequences of a change in CREB function, or in an upstream pathway, in various brain regions are [thus] likely to be multifaceted and difficult to predict a priori.”*

Within the NAcc, for example, genes positively identified for upregulation by CREB include proenkephalin (*Penk1*), prodynorphin (*Pdyn*), and c-Fos (*Fos*) [2, 17, 72, 118], however, evidence supports a much broader extent of influence [71, 72], be it directly or indirectly via IEG induction. Psychostimulants have been shown to induce preprodynorphin mRNA in the NAcc through a CREB-mediated mechanism, and subsequently, these data have been used to establish an increasing recognition for dynorphin in its contribution to the expression of sensitization [90]. Different CREB gene targets are identifed in differing central nuclei, although investitagor bias means that often gene targets are not consistently examined across nuclei. For example, Olson and colleagues [83] only examined GluR1 and TH genes as they are both known to contribute to drug reward in the VTA, with similar practises concerning opioids in the NAcc [17, 93, 108]. 

## CONTROLLING CREB EXPRESSION: CONVENTIONAL MODELS 

The collective in situ and immunoblotting data indicated that CREB and/or activated CREB are regulated by various drugs of abuse within brain regions implicated in addiction. These data raised the question as to the precise role for CREB in mediating addiction-related behaviours To this end, novel transgenic techniques were employed to knockout CREB and observe functional implications. The most obvious beginning was deletion of the DNA binding domain and all of the leucine zipper on the *Creb* gene, producing CREB-null mice (*Creb*^null^) devoid of central and peripheral functional isoforms of CREB through inability to dimerise or bind to DNA (at the CRE site). Null mice were observed to have a reduced birth weight (70%) relative to wild type mice, were cyanotic, and died immediately after birth (perinatally) from respiratory distress (pulmonary atelectasis) [71]. The mutant also exhibited other developmental abnormalities and phenotype including a birth rate less than Mendelian frequency, hypoplasia/atrophy of the corpus callosum and anterior commissure, a markedly reduced thymic cellularity affecting all developmental stages of the αβ-T cell lineage, and upregulated CREM in the hippocampus and other forebrain regions; in contrast, wild type expression of CREM is predominantly restricted to neuroendocrine neurons [98]. The total deletion of all central and peripheral isoforms of CREB were, despite the upregulation in CREM (which possibly allowed the mutant to reach parturition), the likely cause for these apparently diverse defects. Given the contribution of CREB to neuronal growth, plasticity and survival mediated by the neurotrophins including NGF, BDNF and NT-3 [63, 99, 114, 121], the impact of total CREB deletion was ubiquitious, substantially impairing normal organ and physiological development. It has been recently shown that mice with a null mutation for *Creb* restricted to central regions do not show the excessive apoptosis witnessed by peripheral *Creb* null mutation [63], driving subsequent studies into central-specific CREB recombination, discussed later.

## DISCOVERY OF A NOVEL CREB ISOFORM

An early attempt to create a CREB-null mutant led to the development of the now widely recognised *Creb1*^αδ^ mutant. Hummler and colleagues [47] created mutant mice by embryonic stem cell homologous recombination, inserting a promoterless Neo construct into the second exon of the *Creb1* gene, which harbours the first ATG codon [69]. They observed that the proportion of mutant mice surviving was down on expected Mendelian outcome, although adult mutants demonstrated a normal phenotype (measuring for ataxia/motor disorders/nociception/shock response) with no histological or morphological deficits [13]. Blendy and colleagues [10] mused that the healthy disposition of mutants was puzzling because CRE-mediated gluconeogenic enzyme expression (critical for perinatal surivival) should be attenuated by the mutation, and that based on previous evidence of CREB in the pituitary, growth should be retarded. Indeed, while ATF-1 levels were unaffected in the mutants, RNA isoforms of CREM were upregulated (including the activator (CREMτ) and repressor (CREMα, CREMβ) isoforms, which lack an activator-specific exon), and a novel form of CREB, CREB-β, was observed to be upregulated [115]. CREB-β carried a molecular weight of 40kDa and was identical to CREB-δ (the prevalent CREB isoform) except for a deletion of 40 residues of the Q-domain; its upregulation was 6-fold in the brain and 4-fold in the liver, with upregulation also in the testes [10]. CREM isoforms, previously shown to be expressed in (or constrained to) neuroendocrine nuclei, now became expressed ubiquitously throughout the brain [10]. Such mutants have been described as carrying a hypomorphic CREB allele or as being haplodeficient in CREB [87], and a great deal of experimental work has been conducted using this model.

## ADDICTION STUDIES USING MICE HYPOMORPHIC FOR CREB

The balance of studies examining the impact of CREB knockdown in the context of addiction have done so with the CREB^αδ^ paradigm, probably because the model does not require time-consuming preparation and development, whilst still providing an interesting experimental subject. Such mutants do express higher than normal CREB-β levels, although studies have suggested [47, 66] and recently shown that total CREB activity is reduced approximately 90% throughout the brain (cortex, cerebellum, sub-cortical nuclei) [115]. Clearly, the peripheral expression of CREB-β, though impaired, is enough to propagate required developmental homeostatic mechanisms in these mutants. The studies shown in Table **2** demonstrate a complex phenotype of this CREB knockout model. Numerous studies provide evidence that mutants have attenuated behavioral response to morphine withdrawal [66, 112, 115]. These mutants are basally more anxious than WT [87], which was demonstrated to impact upon EtOH (drug) preference rather than natural reward preference. Interestingly, whilst some studies demonstrate that cocaine and morphine earn a similar salience as well as exerting similar behavioural influence in mutants as wild types [59, 66, 112] others provide evidence to the contrary, or at least which hinders us in drawing any compelling conclusions [115, 117]. Moreover, Walters and colleagues [116] recently demonstrated that although 1mg/kg nicotine was rewarding to WT but not mutants, 2mg/kg nicotine elicited an aversive response in both mutants and WT’s, underlying the difficulty and complexity associated with interpretation of these results.

Collectively, these data emphasise the differential and unpredictable effect of diminished CREB throughout the CNS on behaviour and gene transcription [66, 87, 118], including various memory and learning conditions (for example, deficits observed in some but not all long term memory tests) [13, 34, 39]. No doubt complicating such results is the upregulation of CREM throughout the CNS so that it no longer distributes primarily in neuroendocrine-associated nuclei, as well as the upregulation in CREB-β [10], affecting the consistent reporting of affective and cognitive deficits. Whist this model is a step toward ascertaining the contribution of CREB to addiction, it is inferior to recent spatiotemporal models where CREB deletion is total, and the subject’s system has little time or scope to compensate for the knockout. Given the problems encountered with the peripheral knockdown of CREB, various novel models have been employed to target central expression of CREB, and where possible, to selectively target brain nuclei.

## CREB AND DOMINANT-NEGATIVE CREB OVEREXPRESSION USING HSV

One such model is herpes simplex virus (HSV)-mediated overexpression of CREB in specific brain nuclei or, in order to gain a local knockdown of CREB, the dominant-negative isoform mCREB (mutant CREB). Mutant CREB (mCREB) contains a serine-to-alanine substitution at position 133, eliminating the cAMP-dependent protein kinase phosphorylation site but maintaining charge balance. Subsequently, whilst mCREB can still bind to cAMP responsive elements (CREs), it inhibits active CREB by occupying the CRE and preventing access by wild-type CREB and other CRE-binding proteins [98, 106]. The HSV-(m)CREB system achieves maximal expression of virally-encoded transgenes by 24 hours post-injection, which persists for three to four days before dissipating to trace or zero expression by day seven post-injection [83]. A number of recent studies have examined the impact of HSV overexpression of (m)CREB in discrete regions of the CNS upon phenotype, shown in Table **3**. The balance of these studies have as a key endpoint the impact of (m)CREB overexpression upon the rewarding effects of cocaine or morphine [7, 17, 93], finding that CREB overexpression in the NAcc shell decreases drug reward whilst mCREB enhances drug reward, results that may also apply to natural rewards [7]. Such data suggest that immediately following drug intake, upregulation in accumbal CREB (or increased phosphorylation of CREB) may diminish the salience of further drug administration. This suggests a contribution to either early development of tolerance, or development of an inbuilt safety-mechanism which is activated as the body recovers from drug insult. Other findings using this system drive home the observation that regional CREB or mCREB overexpression has a markedly different impact upon either reward [17, 83] and affective states measured through phenotypic traits of anxiety or depression [7, 24, 93], or of physical dependence [42]. Using recombinant Sindbis pseudovirions to drive constitutively active or dominant negative CREB overexpression in the NAcc, Dong and colleagues [29] demonstrated a recovery or further decrement in MSN excitability, respectively, in a rat model of cocaine bingeing. 

This model is conceptually appealling, and indeed, the overexpression of dominant negative (mCREB) allows investigators to deduce the impact of substantial CREB knockdown in specific brain nuclei. In contrast, numerous studies have suggested that alterations in CREB levels *per se* following cross-temporal drug abuse seem to occur unpredictably, if at all. A far more consistent expression marker is its phosphorylated or active from, pCREB, and the cogent point made by Walters and colleageus [118] applies, “*Given that phosphorylated CREB (pCREB) is the transcriptionally active form of CREB and that a given stimulus might lead to the phosphorylation of only a few dozen molecules of CREB per cell, alterations in total CREB levels achieved by genetic manipulations might or might not lead to significant changes in pCREB depending on the original protein levels present”*. In the balance of the HSV-CREB overexpression studies, experiments are conducted 2-3 days post-injection. Given that CREB (or more accurately, pCREB) expression shows a distinct and no doubt critically relevant temporal regulation following drug abuse stimuli, we are hard pressed to guess at what sort of remodelling effects prolonged CREB exposure may implement in discrete neural regions. Moreover, this overexpression period is unlikely to complement anything wrought upon CREB or pCREB expression by pharmacological or stressor methodology. Drugs of abuse alter pCREB levels directly through kinase pathways, and CREB levels indirectly through gene expression systems; however, viral (HSV) overexpression systems increase CREB directly, without altering pCREB levels directly through endogenously occuring systems. Subsequently, these results are somewhat difficult to interpret in regards of existing literature examining drugs of abuse in wild type mice given a differential method and time course in generating synaptic plasticity and neuroadapatation. Finally, whilst CREB is constitutively expressed in the nucleus, viral overexpression of CREB enters via the cytosol, suggestive of stochastic or unknown levels of trafficking into the nucleus to affect target CRE-binding and subsequent plasticity. Although the extent and kinetics of mCREB dimerisation (with CREB, CREM and ATF-1), CRE-DNA binding and subsequent dimer transcriptional activation potency is not fully known [65], HSV-mCREB overexpression appears to be a promising strategy in the ongoing elucidation of central CREB’s impact in the context of addiction studies.

## TETRACYCLINE-REGULATED TRANSACTIVATION AND CREB ANTISENSE

Another attempt at regulating CREB expression in brain is the tetracycline transactivator system, whereby doxycycline in the drinking water is the “switch” that is coupled to the suppression of transgene expression. CREB overexpression can subsequently be targeted to the brain, with a tetracycline transactivator (tTA) controlled by a 1.8kb neuron-specific enolase (NSE) promoter. Sakai and colleagues [101] crossed mouse lines to generate NSE-tTA TetOP-CREBα bi-transgenic mice, creating a system controlled by NSE, whose expression generates tTA, binding to the TetOP promoter, generating CREBα overexpression. Addition of a tetracycline analogue, doxycycline, binds to a Tet binding pocket on the tTA, which undergoes a conformational change and binds to TetOP in such a way, inhibiting CREBα expression. Their model demonstrated that CREB overexpression in the brain had an inconsistent influence upon expression of other members of the CREB/ATF family (invariably downregulating though), paricularly CREM. CREB overexpression was restricted to the nucleus of cells, predominantly in the striatum, although with some expression observed within the cingulate cortex and hippocampus. In contrast, Pittenger and colleagues used this model to overexpress dominant negative human CREB (KCREB) in either the dorsal striatum (and olfactory tubercle) [91] or dorsal hippocampus (CA1, and striatum/piriform cortex) [92], the former demonstrating a contribution of CREB to procedural learning, the latter, a somewhat subtle phenotype in relation to spatial learning and memory. A major downfall of this system, however, is the time lapse (seven to fourteen days) required to drive corresponding alteration in gene expression, a critical feature regarding (m/K)CREB cytological (dys)regulation.

Antisense models have also been utilised, involving the local knockdown of CREB by infusion of CREB antisense, which binds to *Creb* mRNA thus inhibiting further translation. A small number of studies (Table **4**) employing CREB antisense collectively demonstrate the contribution of CREB to drug-induced behavioural phenotype and mRNA or protein expression. Whilst antisense seems to provide the ‘magic bullet’ to functional gene analysis, difficulty with delivery systems, specificity, toxicity and inconsistent effects are key drivers for its limited adoption in experimentation.

## CONVENTIONAL CREB KNOCKDOWN MODELS: THE CONCLUSION

Underlying the aforementioned ‘conventional’ models of CREB manipulation is that while they have allowed insight, each has a major deficit which may confound interpretation. More recently, through various advances in the field of molecular biology, novel techniques have arisen which confer alternative targeting of the *Creb *gene and control of expression.

## LOOKING FORWARD: REGION-SPECIFIC PROMOTERS DRIVING SPATIOTEMPORAL CREB DELETION

A more recent attempt to selectively target and knockdown CREB from central regions involves a model of region-specific promoters driving the site-specific *Cre* recombinase. This entails the cross-breeding of two strains of mice, one expressing the *Cre* transgene driven by a tissue- or ontogenetic stage-specific promoter, and the other a “floxed” gene (loxP sequences flanking gene of interest for recombination) [110]. Early problems with the model included the time taken to breed the strains, difficulty establishing well-characterised tissue-specific promoters, and the inability to induce *Cre* expression at specific ontogenetic time points [4]. Over time, however, the latter two have been substantially ameliorated. As the expression of *Cre*-recombinase determines the recombination event surrounding the floxed gene, numerous approaches have been taken in addressing its expression profile. Various region-speicific *Cre* promoter models have been developed, including an adipose-specific aP2 enhancer/promoter [6], central- and ontogenetic-specific promoters CamKIIα [18, 19, 51], Emx-1 [21, 38, 49], Foxg1 [43], Nestin [5, 68], a split-polypeptide *Cre* construct [20], tyrosine hydroxylase (TH) neuronal-specific promoter [35], RSV and EF1α promoters [32], rat insulin promoter [95, 96] and CAG promoter [100]. The main design behind such mutants is to induce disruption of all central CREB isoforms through targeting of the 10^th^ *Creb1* exon. Excision of exon 10 from *Creb1* leads to translation of CREB deficient in DNA binding and dimerization functional domains, such that the protein is unstable and thus, there is loss of CREB [68]. The prominent applications of this technology to cellular CREB expression involves tissue-specific promoters, both regional and restricted by cell-type, as well as expression of promoters tied to developmental stages. Example applications of this model are discussed.

## NESTIN AND CAMKIIA DRIVEN *CRE*

The nestin promoter/enhancer is associated with widespread central deletion of the *Creb* gene, as nestin expresses from embryonic stage in all brain regions before separation of neuronal and glial lineages [68], with CREB loss observed in neurons and glial cells. *Creb1*^NesCre^ mice nominally reach 70-80% of control mouse body weight from 2^nd^ post-natal week due to a deficiency in growth hormone. Data from nestin *Creb*-deficient mutants demonstrated no difference in CPP reward to morphine, cocaine or food versus wild type, but that they exhibited attenuated behavioural responses to naloxone-induced morphine withdrawal and had a heightened anxiogenic phenotype [112]. CaMKIIα is an 8.5kb promoter expressed in forebrain neurons from approximately P7 [5, 68] and this model has shown CREB loss in almost eighty percent of forebrain neurons. Indeed in transgenic mice, high CamKIIα *Cre* levels have been demonstrated within the hippocampus, cortex, olfactory bulb and amygdala, and low levels in the striatum, thalamus and hypothalamus. No CamKIIα was detected in the cerebellum [19]. In these mice, CREB protein began to decrease in the hippocampus and cortex by P6 and was observed to be totally deficient in these regions by P15. Together, these mice models provide a robust method for knockdown of all functional CREB isoforms widely throughout the brain at different time points, without complex developmental adaptations.

### When Applied to CREM

CREM deficient mutants were generated to address the question of what role CREM plays in the absence of CREB, given the observation that CREM may upregulate subsequent to CREB knockout. Mantamadiotis and colleagues [68] studied transgenic mice with nestin and CaMKIIα promoter-driven knockout of *Creb1* crossed with *Crem*-deficient mice, generating pre-natal and post-natal CNS-specific protein loss respectively. Mice with prenatal central loss of CREB and CREM died at P1, as they did not suckle for milk; brain structures in these mice were unaffected, but cell density was markedly diminished, largely due to apoptosis; expected Mendelian ratios were observed however. Mice with postnatal central loss of CREB and CREM displayed an abnormal phenotype of retraction of limbs when suspended for 20s, characterising a neurological impairment owing to neurodegeneration, and considerable progressive atrophy of the dorsolateral striatum and CA1 hippocampal neurons; widespread astrogliosis was revealed (glial cell apoptosis) in dorsolateral striatum, CA1, DG and some in amygdala, cortex and thalamus, although a single copy of *Crem* prevented this (suggesting accomodation for loss of CREB); expected Mendelian ratios were observed. Furthermore, *Crem*^-/-^ male mutant mice are known to be sterile, given enhanced apoptosis of post-meioitic germ cells [71], although the mutation does not appear to be as physiologically devastating as does total CREB deletion. CREM null mice have been previously described [67], exhibiting hyperactive and diminished anxiety-like behaviour, however, an addiction-related phenotype remains to be characterised for this model.

## DRD1A DRIVEN *Cre*

Other attempts at promoter-driven CREB deletion have aimed to target brain nuclei selectively, regions critical to the study of addiction. A D1A receptor gene promoter (Drd1a) was employed via 140kb YAC expression vector for *Cre* recombinase, generating a *Cre* expression profile predominantly constrained to the CPu, NAcc and OT, but with some expression observed in layer VI of the cortex, CA2 of the hippocampus and within thalamic nuclei. Also noted was neurodegeneration within the dorsolateral striatum when mutants were double crossed with CREM-deficient mice [68]; further phenotypic analysis is warranted in this model.

## DARPP-*32* DRIVEN *Cre* 

Dopamine and cAMP-regulated phosphoprotein molecular weight 32 kDA (DARPP-32) bears multiple regulatory phosphorylation sites, and acts as an important component in the phosphorylation profile of various cytosolic and membrane-associated proteins. Indeed, in its activated state as pThr^34^-DARPP-32, it becomes a robust inhibitor of protein phosphatase 1 (PP-1), a substrate responsible for de-phosphorylation of numerous cellular targets. DARPP-32 is highly concentrated in medium spiny neurons of the CPu and NAcc (neostriatal neurons), the olfactory tubercule, and lightly in the BNST and amygdala [40, 78]. A novel model of DARPP-32 promoter-driven *Cre *[12] will provide a conduit for the selective knockdown of CREB, allowing the elucidation of the contribution of CREB within the mesolimbic system; demonstrated in Fig. (**2**). Importantly, the expression of DARPP32 driven *Cre* in this model does not commence until 4-5 weeks after birth [12], ensuring a lack of potential developmental compensation.

## Emx1 DRIVEN *Cre*

A final model worth mentioning involves *Emx1* (empty spiracles homologue 1), a homeobox-containing gene which is specifically expressed in the developing (from E10) telencephalic cortex through adulthood [49]. Emx1 is involved in encoding transcription factors, and found in both proliferating and post-mitotic neurons of the cerebral cortex, and may thus be involved in the initiation and maintenance of the neuronal phenotype [21]. Evidence supports its restriction to excitatory pyramidal neurons (layers II-VI, but not layer I) colocalising with glutamate (99.1%) rather than GABA (0.9%), as well as astrocytes and oligodentrocytes of most pallidal structures (vertebrate cerebral cortex primordium) including the hippocampus, neocortex, piriform cortex, endopiriform nucleus and lateral aspects of the amygdala [21, 38, 49]. Within the neocortex and hippocampus, approximately 88% of cells underwent recombination in total [38]. This model is important, as it allows the study of CREB’s contribution to corticostriatal and corticotegmental glutamatergic pathways, both of which have both been strongly implicated in the development of addiction related behaviours, and is demonstrated in Fig. (**2**).

## LOOKING FORWARD: REGION-SPECIFIC VIRAL VECTOR KNOCKDOWN OF CREB

Whilst the promoter-driven strategy for central CREB deletion is good, some of these approaches are still germline in nature, thus suggestive that various difficult-to-identify compensatory adaptations take place in the organism, offsetting the accuracy of experimental results. Correspondingly, investigators have also turned to plasmid based transgene expression systems, however, these tend to be influenced by chromatin position effects and may result in ectopic or mosaic expression. Such plasmid-based transgenes, containing only a few regulatory elements from the gene of interest, often result in unnatural configurations and generally produce highly variable transgene expression patterns [19]. Consequently, many transgenic lines need to be analysed to find one line with the appropriate pattern of expression. A more appropriate and attractive approach used by investigators is that of viral vector based *Cre* delivery systems. This strategy is useful when the phenotype of the total knockout is lethal (embryological mutation, as is the case with CREB), when there are compensatory effects during development (in regard to other gene expression), or when total gene inactivation results in pleiotropic effects involving complex physiological interactions. Viral-vector delivered *Cre* recombinase provides a spatiotemporal model for knocking out “floxed” genes in specific nuclei through stereotaxic injection targeting a brain region or cell group. This novel approach to generating conditional knockouts has been validated on numerous occasions, many involving Rosa 26 Reporter (Rosa26) mice or β-galactosidase expression systems [1, 4, 50, 89, 119]. 

## EMPLOYING THE APPROPRIATE VIRUS

Two widely used virus strains for this purpose are the lentivirus and adeno-associated virus. Lentiviruses (LV) are enveloped RNA viruses that belong to the family of complex retroviruses. In contrast to prototypic retroviruses, such as murine leukemia virus, lentiviruses are able to transduce both dividing and nondividing cells, including neuronal and to a lesser extent glial (astrocyte) recombination. Adeno-associated viral (AAV) vectors induce a substantially lower immunogenic dose than its predecessors, given the total removal of viral genome. AAV is selective toward neuronal recombination *in vivo*, with evidence that it does not recombine in glial cells [50]. Optimal expression is usually 2-4 weeks following injection. Given that AAV and LV are useful in mediating gene delivery and stable transduction to both dividing and non-dividing cells, whilst not inducing an immune response, they provide a valuable role in CNS recombination. AAV vectors are typically limited to accepting only short inserts of up to 5kb [1], however a high capacity adenoviral vector expressing *Cre* recombinase has been described [4]. In this case, all viral coding sequences have been deleted from the viral genome, reducing the expression vector’s toxicity and immunogenicity, whilst increasing the capacity for introduction of heterologous DNA to 36kB, facilitating increased flexibility. Viral-delivered *Cre* can be toxic, likely owing to mammalian pseudo-loxP sequence homology [1, 89], where heightened *Cre *can reduce proliferation [107], induce chromosomal abberation and exchanges with sister chromatids [64], and cause neural (brain) cavities. Chronic high-level expression of *Cre *in spermatids of transgenic mice has also been demonstrated to generate male sterility [102]. This has been addressed by using lower *Cre* doses, whilst still achieving substantial recombination [1], or use of self-deleting viral vectors. The latter has been pioneered with lentiviral delivery of *Cre*, such that the U3 untranslated region (3’ LTR) of the LV vector has a *Cre* insert which expresses only once incorporated into the mammalian genome, self-removing from the genome as *Cre* concentration rises [89, 107]. In the context of the study of addiction, application of this model to selectively target brain regions critical to the development of addiction will prove valuable in determining the contribution of CREB to various pathways of the midbrain and forebrain.

## FUTURE CREB KNOCKDOWN MODELS: THE CONCLUSION

These spatiotemporal paradigms are important indeed, critical, to more efficacious targeting of CREB knockdown in specific cells as well as nuclei. This can better assist in diagnosis of the diverse role CREB plays in neuronal signalling, contributing to addictive behaviours and / or how this phenotype relates to molecular signalling pathways, when both are used in unison. As these models are more widely adopted over subsequent years, the data on the contribution of CREB to the development and expression of addiction should be more reliable, and help us better understand its biological function.

## CONCLUSIONS

This review has briefly discussed the relevance of CREB as a critical biological substrate for the development of addiction, and examined a variety of drug contexts and paradigms with wild type subjects demonstrating its diverse and complex expression pattern. Earlier models, which endeavoured to remove peripheral or central CREB isoforms to determine its impact upon addiction more closely, were found to be in various ways deficient and inadequate for robust and consistent reporting. More recently, spatiotemporal CREB deletion models have provided an illuminating and exciting way forward for the study of total central CREB deletion both temporally, and spatially within the brain, allowing investigators to better characterise precisely the contribution of CREB to the development and / or persistence of the addicted state.

## Figures and Tables

**Fig. (1).CREB signalling pathways.**
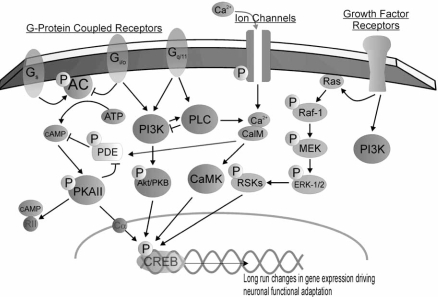
The schematic provides a brief overview of some major intracellular transduction signalling cascades involved in the activation of CREB by phosphorylation at Ser^133^. Synaptic plasticity associated with addiction results from long-term cellular change derived from activation of CREB. Prominent examples of neurochemicals involved in addiction and their complementary receptors, are subsequently indicated. Gs:Adenosine A1/2, Dopamine D1/5; Gi/o: Dopamine D2/3/4, GABA-B; Gq/11: Glutamate (metabotropic) mGluR1/5; Ion Channels: Glutamate NMDA/AMPA GluR1R/Kai-nate, L-Type VGCC; Growth Factor Receptors: EGF/NGF/BDNF TrkA, TrkB. Abbreviations: AC,adenylate cyclase; CalM, calmo-dulin; CaMK, calcium/calmodulin-dependent protein kinase; ERK 1/2, extracellular signal regulated kinase;MEK, MAPK and ERK kinase; PDE, phosphodiesterase; PLC, phospholipase C; PI3K, phosphoinositide-3 kinase; RSK, ribosomal-S6 kinase.

**Fig. (2).CREB knockout circuitry.**
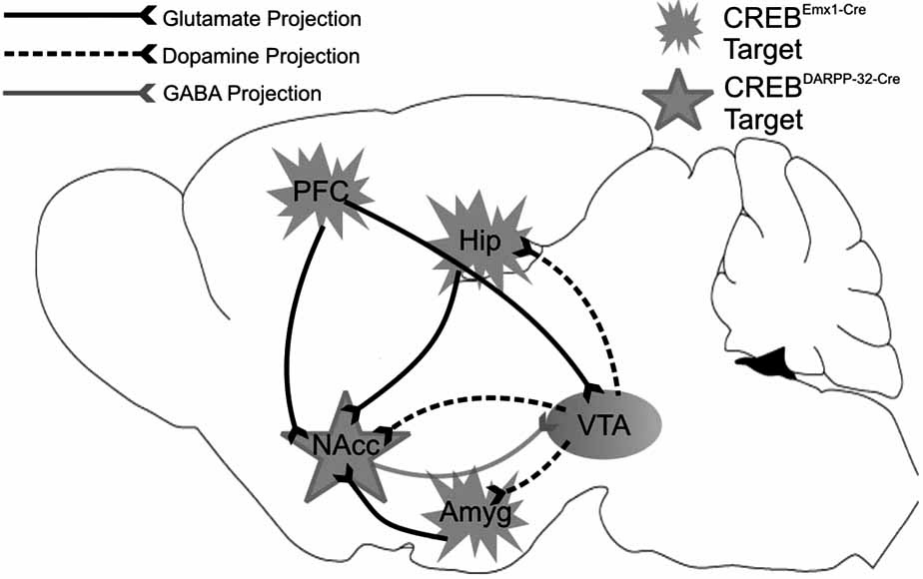
Key nuclei of the limbic circuit are demonstrated in the schematic, as well as their neurochemical projections. All regions confer a salience to the development of addiction, across conditioning (amygdala), learning and memory (hippocampus) and reward and reinforcement (NAcc,VTA, PFC). As mentioned in the main text, Emx1-driven Cre would knockout CREB from the regions indicated, although a major knockdown should be observed in the PFC, thus impacting upon glutamatergic projections to the NAcc and VTA. DARPP-32-driven Cre would knockout CREB from MSNs of the NAcc, allowing us to address the impact upon striatal outflow. Viral vector strategies could allow the specific knockdown of CREB from individual nuclei listed on the schematic. Abbreviations: Amyg, amygdala; Hip, hippocampus; NAcc,nucleus accumbens; PFC, prefrontal cortex; VTA, ventral tegmental area.

**Table 1.** CREB Involvement in Addiction

STUDY	DRUG & TREATMENT SCHEDULE	DELAY TO NEURO- CHEMICAL ANALYSIS^	pCREB EXPRESSION EFFECT
[76]	EtOH Chronic, drinking	0 hour (on final access day)	Decreased in NAcc shell, no change in NAcc core, FC nor CeA
[84]	EtOH Chronic, liquid-diet; Withdrawal	0 hour (on final access day) or 24 hour (withdrawal)	Decreased in CG, no change in cortex*;Change in all regions to withdrawal
[86]	EtOH Chronic, liquid-diet; Withdrawal	0 hour (on final access day) or 24 hour(withdrawal)	No changes in CeA, MeA, nor BLA; Change in all regions to withdrawal
[122]	EtOH Acute, ip; Chronic, liquid-diet	15 minutes, 1 & 6 hour: acute; 0 hour:chronic †	Increase in cerebellum (acute, max at 15 min) or no change (chronic)
[123]	EtOH Acute, ip; Chronic, liquid-diet	5, 15, 30 minutes: acute; 30 minutes: chronic	Increase (acute, max at 30 min) or decrease (chronic) in cerebellum
[41]	Morphine Acute, sc; Chronic or precipitated withdrawal, pellet	1 hour: acute; 24 hour: chronic	Increase (withdrawal), decrease (acute) or no change (chronic) in LC
[74]	Morphine chronic, sensitised, ip; withdrawal & challenge	90 minutes: 3d & 14d withdrawal †	Decrease in NAcc and VP (3d and 14d withdrawal, following challenge)
[44]	Nicotine Acute, repeated, ip	0-6 hour	Increase in adrenal medulla (from 30 min)
[54]	Nicotine Repeated, ip	1 hour	Increase in adrenal medulla
[85]	Nicotine Chronic, withdrawal, ip	1 hour: chronic; 18 hour: withdrawal	Decrease in CG, ParC, PiriC, MeA and BLA (withdrawal) or no change (chronic); No change in FC nor CeA
[94]	Nicotine Acute & chronic withdrawal	1, 18 hour	Decrease in NAcc shell (chronic 18 hour withdrawal) or no change; no change in core
[26]	Amphetamine Acute, chronic, sensitised, ip	1 hour: acute & chronic; 16 hour: chronic	Increase in striatum (sensitised was lower than acute)
[36]	Amphetamine Acute, ip	15 minutes	Increase in striatum
[56]	Amphetamine Acute, ip	2 hour	Increase in striatum
[111]	Amphetamine Acute, chronic, sensitised, ip	2 hour	Increase in striatum (chronic,sensitised)
[75]	Methamphetamine Chronic, sensitised, sc	3d or 14d withdrawal	No change in VTA (3d & 14d); Decreased in NAcc and VP (3d & 14d); Increase (3d) or no change (14d) in FC

^ = time animal was killed after last drug exposure; time periods at which pCREB expression was measured

* = frontal cortex, piriform cortex, parietal cortex

† = extrapolated [from paper]

**Table 2.** Characterisation of the CREB^αδ^Model

STUDY	DRUG EXPOSURE & CONDTION	CREB EXPRESSION EFFECT	PHENOTYPIC EFFECT
[87]	EtOH solution (chronic ?); Sucrose solution (natural reward)EtOH (acute)	Mutant p/CREB was decreased 40% through extended amygdala & cortex; Acute EtOH increased pCREB in the CeA & MeA in WT & mutants	Mutants had higher preference for EtOH but not sucrose; Mutants were more anxious than WT; Acute EtOH was anxiolytic in WT & mutant
[59]	Cocaine CPP model; Stress (FST)	In cocaine-pretreated WT, stress challenge increased pCREB in NAcc, whereas cocaine challenge increased pCREB in amygdala and VTA	Stress didn’t induce reinstatement in cocaine pre-treated mutants, as it did in WT; Cocaine induced reinstatement in cocaine-pretreated mutants and WT
[39]	Fear conditioning (foot shock); Spatial learning (MWM);		Mutants had impaired SR & LR cued and contextualfear conditioning response (associative-learning response),but normal spatial learning/memory
[34]	Fear conditioning (foot shock); Spatial learning / reference memory (MWM); LTP (CA1, DG)		No deficits were observed retention of freezing memory in mutants, nor LTP measures; essentially normal pheotype observed in spatial learning/memory test
[112]	Morphine, cocaine & food CPP model; Naloxone-induced morphine withdrawal		Rewarding effects of morphine, cocaine & food were no different in mutants and WT; mutants had attenuated behavioural response to naloxone-induced withdrawal
[115]	Morphine (chronic); Morphine & cocaine CPP model		Mutants had attenuated behavioural response to chronic morphine withdrawal; Mutants had enhanced response to reinforcing properties of cocaine but not morphine in CPP paradigm; Mutants had enhanced sensitised locomotor behaviours to cocaine
[66]	Morphine (acute, chronic); Naloxone-induced morphine withdrawal Stress (?)		Mutants had attenuated behavioural response to chronic morphine naloxone-induced withdrawal; Mutants had similar acute morphine-induced analgesia, locomotor activity and behaviorual response to stress as WT
[117]	Morphine CPP model (low, high dose)		High dose morphine increased CPP reward and locomotor activity in mutants; Low dose morphine had decreased reward in mutants but unchanged locomotor activity vs WTs;

**Table 3.** HSV-(m)CREB Overexpression Models

STUDY	REGION OF OVEREXPRESSION	DRUG EXPOSURE / PARADIGM	REWARD / PREFERENCE EFFECT
[17]	NAcc Shell, Core	Cocaine CPP model	mCREB in the shell but not core increased preference; CREB in the shell decreased preference
[7]	NAcc Shell	Morphine, sucrose CPP model	mCREB increased morphine and sugar(natural reward) preference; CREB decreased morphine and sugar preference
[24]	Dentate gyrus (DG), CA1 pyramidal layer (CA1), Pre-frontal cortex (PFC)	Antidepressents in a learned helplessness (LH) & Forced swim test (FST) model	Not tested
[42]	Locus Coeruleus	Precipitated morphine withdrawal behaviours	Not tested
[83]	Rostral & caudal VTA, substantia nigra	Cocaine, morphine CPP model	Rostral VTA CREB enhanced drug preference, but mCREB made drug aversive; caudal VTA CREB made drug aversive, but mCREB enhanced durg preference; CREB/mCREB in substantia nigra had no effect on preference; high dose morphine enhanced preference regardless of CREB/mCREB injections in rostral or caudal VTA
[93]	NAcc (shell)	Cocaine CPP model; Forced swim test (FST)	mCREB increased preference, CREB decreased preference

**Table 4.** CREB Antisense Models

STUDY	REGION of ANTISENSE TARGET	DRUG EXPOSURE / PARADIGM	GENOMIC EFFECT	PHENOTYPIC EFFECT
[56]	Striatum	Amphetamine	Antisense inhibited striatal c-fos mRNA unpregulation by amphetamine	
[62]	Locus coeruleus	Morphine; Naloxone-induced withdrawal	Antisense blocked morphine-induced AC VIII and TH but not PKA type II nor Giα upregulation	Antisense attenuated some naloxone-induced withdrawal behaviours
[2]	Lateral CPu	Cocaine	Antisense blocked cocaine-induced CREB, c-Fos, FosB, ΔFosB and prodynorph in mRNA upregualtion;	Antisense enhanced locomotor activity in control rats (saline) but didn’t change stereotypy induced by chronic cocaine
[25]	NAcc core or shell (bilateral)	Cocaine	Antisense reduced regional CREB and BDNF expression; produced transient reduction in reinforcing property of cocaine and reinforcement threshold	
[120]	NAcc (unilateral)	Cocaine 5d injections or mini-pump infusions;killed 18hr later	CRE IR was decreased in the NAcc by 40%; a [time-dependent] reversible decrease in Giα and PKA-C subunit expression by 21% and 27% respectively in the NAcc; Attenuated c-Fos induction by acute cocaine; Didn’t affect numerous other signal transduction pathways, including CaMKII-α /β, PKC-β/γ , Goα , Gβ, PLC-δ /γ or PI3K	

## ABBREVIATIONS

ATF-1: = Activating transcription factor-1
CaMK: = Ca^2+^/calmodulin protein kinase
CREB: = cAMP responsive element binding protein
CREM: = cAMP responsive element modulatory protein
LC: = Locus coeruleus
METH: = Methamphetamine
NAcc: = Nucleus accumbens
PDE: = Phosphodiesterase
PVN: = paraventricular nucleus of the hypothalamus;
